# Severe Thrombocytopenia Two Weeks Following Immunization with the Janssen Ad26.CoV2.S Vaccine

**DOI:** 10.1155/2022/7208401

**Published:** 2022-07-27

**Authors:** Bana Antonios, Markie Zimmer, Emma Herrman, Ramona Berghea

**Affiliations:** Department of Internal Medicine, William Beaumont Hospital, Royal Oak, MI, USA

## Abstract

Immune thrombocytopenia (ITP) has been associated with immunizations with various proposed mechanisms, including overactivation of the immune system and production of antibodies against circulating platelets. ITP has also been associated with several viral infections, including HCV, HIV, and most recently, active SARS-CoV-2 infection. Here, we present a case of a 52-year-old male with no past medical history who sought evaluation with his primary care physician for upper and lower extremity ecchymosis of one week duration. Outpatient laboratory studies were notable for severe isolated thrombocytopenia with platelet count of 8 × 10^^^9/L. Interestingly, he received the Johnson and Johnson COVID-19 vaccine 16 days prior to his presentation. Clinical work up and laboratory investigations led to the diagnosis of ITP.

## 1. Introduction

In February 2021, the Janssen COVID-19 immunization by Johnson and Johnson pharmaceutical company was FDA-approved as a single-dose intramuscular immunization for adults over 18 years of age [1]. By April 2021, 7.98 million doses had been administered with high efficacy [2]. However, the vaccine adverse events reporting system reported 13,725 cases of arterial or venous thrombosis along with thrombocytopenia, a new hematological condition initially known as vaccine-induced thrombotic thrombocytopenia (VITT), now known as thrombotic thrombocytopenic syndrome (TTS). A diagnosis is established in the setting of recent immunization (4 to 30 days prior), venous or arterial thrombosis, thrombocytopenia, and positive antibodies directed against platelet factor-4 (PF4) [2, 3].

To our knowledge, isolated ITP without any evidence of arterial or venous thrombosis has been reported with mRNA COVID-19 immunizations, i.e., Pfizer and Moderna. Only one case has been published describing isolated thrombocytopenia following the Johnson and Johnson vaccine.

## 2. Case Presentation

A 52-year-old male with no past medical history sought evaluation with his primary care physician for upper and lower extremity ecchymosis of one week duration. Outpatient laboratory studies were notable for severe thrombocytopenia with platelet count of 8 × 10^^^9/L, and he was ultimately referred to the emergency department for hospital admission. He denied any recent flu-like symptoms, gingival bleeding, epistaxis, headaches, visual changes, abdominal pain, shortness of breath, leg pain, or swelling. He did not take any medication other than occasional nonsteroidal anti-inflammatory drugs for chronic back pain. He also denied any family history of bleeding or autoimmune disorders. Interestingly, the patient mentioned that he recently received the Janssen Ad26.CoV2.S immunization 16 days prior to his hospital admission.

Initial workup in the emergency department revealed a leukocyte count of 6.8 10^^^9/L (normal range 3.5–10.1 10^^^9/L), hemoglobin 150 g/L (normal range 130–170 g/L), hematocrit 0.45 (normal range 0.40–0.5), MCV 92 *μ*m^3 (normal range 80–100 *μ*m^3), MCH 31 pg/cell (normal range 28–33 pg/cell), MCHC 31 g/dL (normal range 32–36 g/dL), RDW CV 12% (normal range 12–15%), and platelet *f* 8 x 10^^^9/L (normal range 150–400 x 10^^^9/L). The baseline platelet count was 190 x 10^^^9/L in years prior. Serum chemistries, including electrolytes, renal function, and hepatic function were all unremarkable. Total bilirubin was 5.13 *μ*mol/L (normal range 5.13–20.5 *μ*mol/L), with direct 1.7 *μ*mol/L (normal range 0.0–6.8 *μ*mol/L). The reticulocyte count was 86 × 10^^^9/L (normal range 21–100 10^^^9/L) and immature reticulocyte fraction was 0.07 (normal range 0.01–0.16). The prothrombin time (PT) was 12.0 seconds (normal range 9.2–13.5 seconds), activated partial thromboplastin time (aPTT) was 29.9 seconds (normal range 25–38 seconds), and international normalized ratio (INR) was 1.1. D-dimer was 413 mg FEU/L (normal range 0–499 mg FEU/L) and fibrinogen 4736 *μ*mol/L (normal range 3420–6480 *μ*mol/L). Vitamin B12 and folate levels were within normal limits and iron studies were not available. HIV, HCV, ANA, TSH, and *H. pylori* were all unremarkable. Peripheral smear revealed normal morphology of neutrophils and monocytes, with small and mature lymphocytes; normocytic normochromic red blood cells without significant anisopoikilocytosis; severe thrombocytopenia with normal platelet morphology. ELISA results of IgG antibodies against platelet factor-4 (PF4) were negative with 0.131 OD (optical density normal range <0.400 OD). Abdominal ultrasound revealed patent hepatic vasculature and unremarkable liver and spleen, with spleen measuring 11.4 cm in length.

The differential diagnosis for severe thrombocytopenia is broad, particularly in a patient with no known prior medical history. Serum chemistries were unremarkable, including a disseminated intravascular coagulation (DIC) screen, making DIC unlikely, further supported by lack of hemolysis on peripheral smear. He was not infectious, ruling out sepsis. Viral causes, such as SARS-CoV-2, HIV, and HCV were negative. Nutritional investigations, including vitamin B12 and folate, were within normal limits. He had no history of alcohol use disorder, making toxic marrow suppression unlikely. Abdominal imaging revealed a normal-sized spleen and patent hepatic vasculature, ruling out hepatic vasculopathy or hypersplenism. Additionally, his coagulation studies were within normal limits. ANA was negative, and with his age and no personal or family history of autoimmune conditions or other symptoms, systemic lupus erythematosus was effectively ruled out.

Thus, ITP, thrombotic thrombocytopenic purpura (TTP), heparin-induced thrombocytopenia (HIT), and drug side effects were the highest on the remaining differential diagnosis. PLASMIC score, which predicts ADAMTS13 deficiency in TTP, was 5 at admission, indicating an intermediate risk for TTP. However, ADAMTS13 level was not checked, nor was plasmapheresis initiated. Though he had no exposure to heparin, the pretest scoring system for HIT, so-called 4 T score was 3, corresponding to a low HIT probability and antibodies directed against PF4 were negative. There was concern for TTS in the setting of recent vaccination against COVID-19 with the Janssen Ad26.CoV2.S vaccine. There was negative HPF4 serology and no evidence of thrombosis, effectively ruling out VITT and finalizing a diagnosis of ITP.

Hematology/oncology consultation was obtained from the emergency department. He was transfused with one unit of apheresed platelets with a minimum of 2.9 × 10^^^11 platelets and started on dexamethasone 40 mg daily for four days in the setting of high suspicion for ITP. Heparin products were avoided during the investigations.

**Figure 1:**
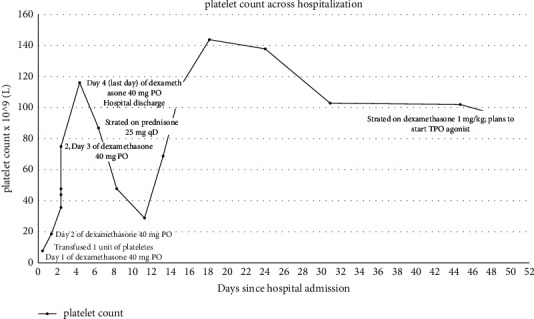
Platelet count across hospitalization.

The patient was discharged from the hospital to complete his last planned dose of dexamethasone 40 mg PO at home that evening. His platelet count across hospitalization is shown in Figure 1.

Based on available outpatient medical records across 48 days since hospital discharge, the patient had close follow-up with his primary care physician and hematology/oncology. Unfortunately, he had relapse of thrombocytopenia and was again started on prednisone with slow taper course, but at the time of this writing, was deemed steroid dependent and Hematology/oncology was planning to initiate a thrombopoietin receptor (TPO) agonist.

## 3. Discussion

Immune thrombocytopenia (ITP) is a rare disease characterized by immune-mediated destruction of platelets leading to thrombocytopenia and increased bleeding risk. Immunizations are a recognized cause of secondary ITP. The proposed mechanism of this entity is molecular mimicry, where the epitope of the antigen shares properties with a self-peptide. Autoantibodies form and target platelet surface glycoproteins, most commonly GPIIb-IIIa and/or GPIb-IX, causing destruction of platelets and subsequent isolated thrombocytopenia [1, 3, 4].

ITP is usually a diagnosis of exclusion. Initial work up usually aims at excluding potential causes such as viral or bacterial infections, most commonly HIV, hepatitis C, and *Helicobacter pylori*. Low B12 and folate levels could also cause thrombocytopenia but is often associated with anemia or leukopenia. Evaluation for underlying autoimmune disease is also important to rule out, such as systemic lupus erythematosus and antiphospholipid syndrome, where thrombocytopenia is a common finding. Physical exam and abdominal imaging assessing for underlying organomegaly is important to exclude liver disease or hypersplenism.

Multiple case reports have now described ITP in patients diagnosed with SARS-CoV-2 infection as well as following administration of the mRNA immunizations—Moderna and Pfizer‐BioNTech BNT16B2b2 [3, 5]. A case series at Cleveland Clinic hospitals noted an incident of 0.34% of COVID-19-induced thrombocytopenia. Reported results concluded response to corticosteroids, as well as the use of thrombopoietin receptor agonists (TPRAs) as second-line treatment [6]. A case series completed only months after Moderna and Pfizer use was widespread amongst the public population described 20 cases of thrombocytopenia within 2 weeks of receiving Moderna or Pfizer vaccines [3].

To our knowledge, ITP has not been reported following the Janssen Ad26.CoV2.S immunization. However, there have been reported cases of thrombosis with thrombocytopenia syndrome following its administration. The term vaccine-induced thrombotic thrombocytopenia (VITT) was proposed to describe this novel entity to avoid confusion with HIT [7] but has since been renamed thrombosis with TTS. TTS is rare and characterized by arterial or venous thrombosis in the presence of new-onset thrombocytopenia. In one case series, 12 of the 15 thrombotic events that occurred were a cerebral venous sinus thrombosis (CVST). Administration of the Janssen Ad26.CoV2.S vaccine was initially suspended after 6 patients developed CVST [7]. Interestingly, CVST has been observed following the AstraZeneca immunization approved in Europe as well, but has not been observed following administration of the mRNA immunizations. AstraZeneca and Janssen Ad26.CoV2.S have similar mechanisms, with AstraZeneca being a chimpanzee adenoviral vector, and Janssen Ad26.CoV2.S being a human adenoviral vector. In case reports of those who developed TTS following AstraZeneca administration, it appeared via a similar mechanism as heparin-induced thrombocytopenia, with elevated antibodies directed against PF4 despite lack of recent administration of heparin [8]. Antibodies directed against PF4 were included in our work up despite the fact that our patient did not have a recent heparin use, given the recently raised awareness of thrombotic thrombocytopenic syndrome associated with certain types of the SARS-CoV-2 vaccines.

General guidelines of ITP treatment vary based upon presentation and severity of thrombocytopenia. In cases presenting with critical bleeding, it is important to rapidly raise the platelets count when it is below 10 × 10^^^9/L with platelet transfusions. However, for ITP cases presenting with minor bleeding and patients who remain asymptomatic, treatment is reserved for platelet count of below 50 × 10^^^9/L and below 20 × 10^^^/L, respectively. Glucocorticoids are the preferable initial therapy, with either a pulse dose of dexamethasone, usually 40 mg daily for four days or methylprednisolone 1 g daily for three days, or 1 mg/kg of prednisone followed by a slow taper. Intravenous immunoglobulin (IVIG) is another option that can increase platelet count rapidly and is generally reserved for cases refractory to glucocorticoids [3, 4]. Bone marrow aspiration is usually reserved for patients over 60 years old to rule out myelodysplastic syndrome or other potential malignancies such as leukemias and aplastic anemia. However, in ITP patients, it is not a requirement for diagnosing but is considered in steroid-resistant cases [8–10].

Another small case series evaluated ITP cases observed within two weeks after administration of mRNA COVID-19 vaccine. Fifteen cases followed the Pfizer-BioNTech version and thirteen cases followed the Moderna version. Most of the patients were treated with glucocorticoids and IVIG. However, as the majority of them required more than one line of treatment, we note that vaccine-induced ITP appears to be resistant to steroids therapy. Unfortunately, the data about these cases are brief and limited, and two patients suffered lethal consequences [11].

To the best of our knowledge, this is an early case of ITP following the administration of the Janssen Ad26.CoV2.S immunization at the time of presentation. There has only been one reported case of isolated thrombocytopenia without thrombosis since. Certainly, there is no definitive evidence to argue that this was not an initial episode of primary ITP coincidentally surrounding the immunization administration. Strikingly, the timeline of events, as well as the thorough workup to exclude other secondary causes, leads us to believe that the Janssen Ad26.CoV2.S immunization caused secondary ITP in this patient.
